# Mortality associated with nonrestorative short sleep or nonrestorative long time-in-bed in middle-aged and older adults

**DOI:** 10.1038/s41598-021-03997-z

**Published:** 2022-01-07

**Authors:** Takuya Yoshiike, Tomohiro Utsumi, Kentaro Matsui, Kentaro Nagao, Kaori Saitoh, Rei Otsuki, Sayaka Aritake-Okada, Masahiro Suzuki, Kenichi Kuriyama

**Affiliations:** 1 Department of Sleep–Wake Disorders, National Center of Neurology and Psychiatry, National Institute of Mental Health, 4-1-1 Ogawa-Higashi, Kodaira, Tokyo 187-8553 Japan; 2 Department of Psychiatry, The Jikei University School of Medicine, Tokyo, Japan; 3 Department of Laboratory Medicine, National Center of Neurology and Psychiatry, National Center Hospital, Tokyo, Japan; 4 Department of Psychiatry, National Center of Neurology and Psychiatry, National Center Hospital, Tokyo, Japan; 5 Department of Psychiatry, Nihon University School of Medicine, Tokyo, Japan; 6 Department of Health Sciences, Saitama Prefectural University, Saitama, Japan

**Keywords:** Health policy, Human behaviour, Ageing, Neurophysiology, Predictive markers

## Abstract

Associations of sleep duration with human health could differ depending on whether sleep is restorative. Using data from 5804 participants of the Sleep Heart Health Study, we examined the longitudinal association of sleep restfulness combined with polysomnography-measured total sleep time (TST) or time in bed (TIB), representing different sleeping behaviors, with all-cause mortality. Among middle-aged adults, compared with restful intermediate TST quartile, the lowest TST quartile with feeling unrested was associated with higher mortality (hazard ratio [HR], 1.54; 95% confidence interval [CI] 1.01–2.33); the highest TST quartile with feeling rested was associated with lower mortality (HR, 0.55; 95% CI 0.32–0.97). Among older adults, the highest TIB quartile with feeling unrested was associated with higher mortality, compared with restful intermediate TIB quartile (HR, 1.57; 95% CI 1.23–2.01). Results suggest a role of restorative sleep in differentiating the effects of sleep duration on health outcomes in midlife and beyond.

## Introduction

Sleep duration is globally recognized as one of the critical determinants of human health. Numerous epidemiological studies have shown the association of self-reported sleep duration with mortality^1–3^. However, subjective habitual sleep time may substantially differ from that recorded objectively^4^, with the latter reflecting homeostatic sleep regulation more objectively. Therefore, objective sleep quantification could provide a better understanding of the relationship between sleep duration and mortality in the general population. Although it is plausible that shorter sleep duration may lead to higher mortality in terms of sleep homeostasis^5–7^, the relationship between longer sleep duration and higher mortality remains a matter of debate. The balance between the supply and demand of sleep could be a key to understanding this relationship. Evidence for objectively recorded sleep patterns suggests that daily sleep requirements steadily decline with increasing age^8^, but sleep opportunity, which also affects sleep homeostasis^9^, remains unchanged. Given that middle-aged adults are more likely to get insufficient sleep than older adults (excess of demand over supply)^10,11^, objective total sleep time (TST) might be more informative than objective time in bed (TIB) particularly in middle-aged adults. In comparison, older adults physiologically require less sleep, while being allowed to allocate more time for sleep (excess of supply over demand)^12,13^. Such excessive bed rest may become detrimental to human health particularly in the elderly^5,12,14,15^. Consequently, objective TST and TIB must be considered separately when assessing the association between sleep duration and mortality.

Although there is no clear measure that could adequately reflect the degree to which one’s sleep is physiologically fulfilled, the sense of restfulness following sleep may have a unique role in determining whether sleep becomes beneficial to human health, particularly when sleep is considered to be a restorative process^16–18^. A decline in sleep restfulness appears to be distinct from other insomnia symptoms (e.g., difficulty initiating or maintaining sleep)^19–21^, and this particular aspect could be indicative of success in the restorative process achieved through sleep. Given that sleep restfulness has been associated with objective sleep stability in prior studies, including the Sleep Heart Health Study (SHHS)^22–24^, it is plausible that a difference in sleep restfulness could have a quantifiable impact on survival probability among those having similar TST or TIB. Accordingly, concurrent measurements of sleep restfulness could aid our understanding of how sleep duration affects mortality.

Using data from the SHHS, a multicenter population-based prospective cohort study^25,26^, we aimed to separately evaluate the effects of TST and TIB, both individually and combined with sleep restfulness, on all-cause mortality in middle-aged and older adults.

## Results

### Participant characteristics

The SHHS cohort included 3128 middle-aged and 2676 older adults with a mean (standard deviation [SD]) age of 54.5 (6.6) and 73.3 (5.7) years at baseline, respectively. Among all participants, there was a steady decline in TST with increasing age, while TIB remained unchanged. Mean scores on sleep restfulness were slightly below 3 among middle-aged adults and increased with age. There was a sharp decline in the weekend-weekday difference in habitual sleep duration and a steady rise in the number of naps with age (Supplementary Fig. 1). Tables 1 and 2 report demographic, health, and sleep characteristics varying across TST and TIB categories for middle-aged adults and for older adults, respectively.

**Table 1:** Baseline demographic, health, and sleep characteristics of middle-aged adults by TST quartile or TIB quartile (n = 3128).

Characteristic	TST			TIB		
	Q1: < 331 min (n = 776)	IQR: 331 to < 414 min (n = 1566)	Q4: ≥ 414 min (n = 786)	Q1: < 400 min (n = 779)	IQR: 400 to < 477 min (n = 1562)	Q4: ≥ 477 min (n = 787)
Age, mean (SD), y	55.0 (6.7)	54.7 (6.5)	53.5 (6.6)	54.3 (6.6)	54.5 (6.6)	54.5 (6.5)
**Race, n (%)**						
Caucasian	599 (77.2)	1302 (83.1)	639 (81.3)	603 (77.4)	1300 (83.2)	637 (80.9)
Other	177 (22.8)	264 (16.9)	147 (18.7)	176 (22.6)	262 (16.8)	150 (19.1)
Women, n (%)	358 (46.1)	772 (49.3)	513 (65.3)	373 (47.9)	790 (50.6)	480 (61.0)
Body mass index, mean (SD)^a^	29.4 (5.8)	28.4 (5.3)	27.8 (5.2)	29.0 (5.7)	28.5 (5.4)	28.1 (5.0)
**Smoking status, n (%)**						
Current	122 (15.7)	187 (11.9)	83 (10.6)	130 (16.7)	184 (11.8)	78 (9.9)
Former	313 (40.3)	656 (41.9)	303 (38.5)	322 (41.3)	633 (40.5)	317 (40.3)
Never	341 (44.0)	723 (46.2)	400 (50.9)	327 (42.0)	745 (47.7)	392 (49.8)
Apnea hypopnea index (4% oxygen desaturation), mean (SD), events/h	10.9 (14.7)	8.8 (13.0)	7.2 (12.2)	9.8 (13.2)	9.0 (13.7)	7.8 (12.5)
Sleep time with saturated oxygen below 80%, mean (SD), % time	0.18 (0.97)	0.23 (2.71)	0.16 (2.33)	0.19 (1.56)	0.23 (2.57)	0.17 (2.34)
Stroke, n (%)	17 (2.2)	16 (1.0)	6 (0.8)	9 (1.2)	22 (1.4)	11 (1.4)
Myocardial infarction, n (%)	36 (4.6)	53 (3.4)	27 (3.4)	39 (5.0)	50 (3.2)	27 (3.4)
Hypertension, n (%)	312 (40.2)	481 (30.7)	200 (25.4)	288 (37.0)	470 (30.1)	235 (29.9)
Diabetes, n (%)	51 (6.6)	74 (4.7)	35 (4.5)	53 (6.8)	65 (4.2)	42 (5.3)
PSG TST, mean (SD), min	287.5 (38.3)	374.3 (23.1)	441.2 (21.6)	306.1 (47.6)	374.9 (42.4)	421.6 (47.2)
PSG TIB, mean (SD), min	376.1 (60.9)	436.3 (37.7)	487.3 (25.1)	356.2 (39.4)	439.4 (21.6)	501.0 (16.4)
Sleep Restfulness score (1–5), mean (SD)	2.6 (1.7)	2.9 (1.1)	3.0 (1.1)	2.9 (1.2)	2.8 (1.1)	2.9 (1.1)
Stage REM sleep, mean (SD), % time	18.5 (7.1)	20.6 (5.6)	22.5 (5.2)	19.6 (6.7)	20.6 (6.0)	21.3 (5.6)
Physical Functioning Score on SF-36, mean (SD)^b^	80.3 (22.4)	84.8 (19.4)	85.1 (18.2)	82.3 (21.3)	84.3 (19.9)	84.1 (18.8)
Antidepressant use, n (%)	56 (7.2)	116 (7.4)	81 (10.3)	50 (6.4)	121 (7.7)	83 (10.5)
Benzodiazepine use, n (%)	27 (3.5)	66 (4.2)	24 (3.1)	22 (2.8)	63 (4.0)	32 (4.1)
Epworth Sleepiness Scale score (0–24), mean (SD)	8.1 (4.6)	8.1 (4.4)	7.8 (4.4)	8.3 (4.7)	8.1 (4.5)	7.6 (4.2)
Number of daytime naps per week, mean (SD)	2.5 (3.4)	2.1 (2.9)	1.8 (3.4)	2.3 (3.1)	2.1 (3.2)	1.9 (3.1)
Weekend-weekday difference in habitual sleep duration, mean (SD), h	0.63 (0.99)	0.60 (0.89)	0.65 (0.94)	0.68 (0.97)	0.61 (0.89)	0.57 (0.95)
Insomnia or poor sleep, n (%)	293 (37.8)	502 (32.1)	244 (31.0)	244 (31.3)	523 (33.5)	273 (34.7)

*SD* standard deviation, *PSG* polysomnography, *TST* total sleep time, *TIB* time in bed, *REM* rapid eye movement.

^a^Body mass index is calculated as weight in kilograms divided by height in meters squared.

^b^SF-36, Short Form 36 Health Survey.

**Table 2:** Baseline demographic, health, and sleep characteristics of older adults by TST quartile or TIB quartile (n = 2676).

Characteristic	TST			TIB		
	Q1: < 310 min (n = 664)	IQR: 310 to < 396 min (n = 1339)	Q4: ≥ 396 min (n = 673)	Q1: < 404 min (n = 667)	IQR: 404 to < 482 min (n = 1336)	Q4: ≥ 482 min (n = 673)
Age, mean (SD), y	74.1 (5.7)	73.2 (5.7)	72.6 (5.6)	73.3 (5.7)	73.1 (5.5)	73.6 (6.1)
**Race, n (%)**						
Caucasian	566 (85.2)	1202 (89.8)	599 (89.0)	585 (87.7)	1198 (89.7)	584 (86.8)
Other	98 (14.8)	137 (10.2)	74 (11.0)	82 (12.3)	138 (10.3)	89 (13.2)
Women, n (%)	310 (46.7)	648 (48.4)	438 (65.1)	328 (49.2)	694 (51.9)	374 (55.6)
Body mass index, mean (SD)^a^	28.0 (5.0)	27.8 (4.6)	27.4 (4.4)	28.0 (4.9)	27.7 (4.4)	27.8 (4.8)
**Smoking status, n (%)**						
Current	45 (6.8)	86 (6.4)	41 (6.1)	44 (6.6)	90 (6.7)	38 (5.6)
Former	339 (51.0)	603 (45.0)	296 (44.0)	327 (49.0)	615 (46.0)	296 (44.0)
Never	280 (42.2)	650 (48.5)	336 (49.9)	295 (44.2)	631 (47.2)	339 (50.4)
Apnea hypopnea index (4% oxygen desaturation), mean (SD), events/h	13.0 (15.6)	11.7 (13.5)	10.2 (12.3)	11.7 (14.3)	11.3 (12.9)	12.3 (15.0)
Sleep time with saturated oxygen below 80%, mean (SD), % time	0.34 (4.03)	0.13 (1.05)	0.16 (1.27)	0.27 (3.90)	0.12 (1.04)	0.26 (1.60)
Stroke, n (%)	52 (7.8)	69 (5.2)	34 (5.1)	50 (7.5)	57 (4.3)	48 (7.1)
Myocardial infarction, n (%)	82 (12.5)	127 (9.5)	61 (9.1)	74 (11.1)	133 (10.0)	63 (9.4)
Hypertension, n (%)	427 (64.3)	724 (54.1)	334 (49.6)	396 (59.4)	715 (53.5)	374 (55.6)
Diabetes, n (%)	102 (15.4)	148 (11.1)	62 (9.2)	90 (13.5)	139 (10.4)	84 (12.5)
PSG TST, mean (SD), min	258.0 (44.8)	354.7 (24.2)	425.4 (22.1)	284.4 (56.8)	358.6 (49.7)	391.8 (58.8)
PSG TIB, mean (SD), min	380.3 (72.4)	440.6 (41.5)	484.4 (27.4)	351.9 (43.9)	445.2 (21.8)	503.6 (14.3)
Sleep Restfulness score (1–5), mean (SD)	2.8 (1.3)	3.2 (1.2)	3.5 (1.2)	3.1 (1.3)	3.1 (1.2)	3.2 (1.2)
Stage REM sleep, mean (SD), % time	17.1 (7.4)	19.4 (6.0)	19.6 (5.6)	18.3 (7.2)	19.1 (6.2)	19.0 (5.8)
Physical Functioning Score on SF-36, mean (SD)^b^	69.6 (26.2)	73.3 (24.0)	74.3 (23.5)	72.8 (24.4)	73.9 (23.7)	69.9 (25.8)
Antidepressant use, n (%)	46 (6.9)	74 (5.5)	47 (7.0)	46 (6.9)	80 (6.0)	41 (6.1)
Benzodiazepine use, n (%)	53 (8.0)	90 (6.7)	48 (7.1)	54 (8.1)	100 (7.5)	37 (5.5)
Epworth Sleepiness Scale score (0–24), mean (SD)	7.6 (4.4)	7.6 (4.2)	7.5 (4.5)	7.8 (4.4)	7.6 (4.2)	7.4 (4.4)
Number of daytime naps per week, mean (SD)	3.8 (4.5)	3.4 (3.6)	2.9 (3.7)	3.7 (4.5)	3.4 (3.8)	3.1 (3.5)
Weekend-weekday difference in habitual sleep duration, mean (SD), h	0.16 (0.63)	0.12 (0.53)	0.15 (0.59)	0.18 (0.57)	0.13 (0.56)	0.12 (0.60)
Insomnia or poor sleep, n (%)	258 (38.9)	432 (32.3)	222 (33.0)	226 (33.9)	461 (34.5)	224 (33.3)

*SD* standard deviation, *PSG* polysomnography, *TST* total sleep time, *TIB* time in bed, *REM* rapid eye movement.

^a^Body mass index is calculated as weight in kilograms divided by height in meters squared.

^b^SF-36, Short Form 36 Health Survey.

### Associations of total sleep time, time in bed, and sleep restfulness with survival

A total of 223 (7.1%) and 991 (37.0%) deaths were reported in middle-aged and older adults over a median (interquartile range [IQR]) follow-up time of 12.3 (11.3–13.5) and 11.3 (8.2–12.2) years, respectively. Among the participants analyzed, 3083 of 3128 middle-aged adults (98.6%) and 2574 of 2676 older adults (96.2%) who survived the first 2 years were included in a sensitivity analysis.

### Middle-aged adults

#### Duration

**Figure 1:**
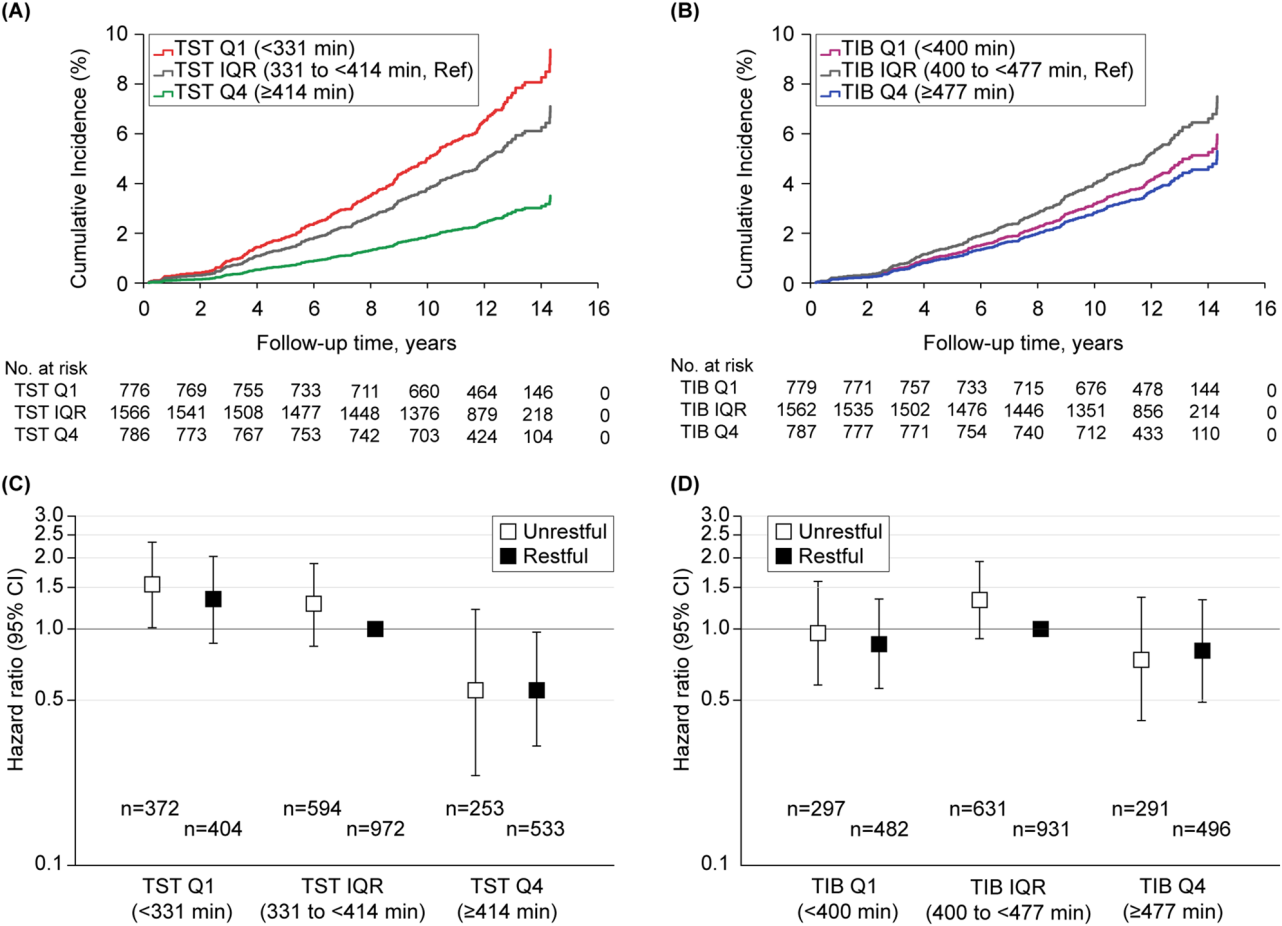
Adjusted Cox regression plots by total sleep time, time in bed, and sleep restfulness for middle-aged adults. Differential cumulative incidences or hazard ratios from the fully adjusted Cox proportional hazard models (Model 3) are shown for middle-aged adults: (**A**) cumulative incidences by TST quartiles; (**B**) cumulative incidences by TIB quartiles; (**C**) hazard ratios by TST quartiles with sleep restfulness; and (**D**) hazard ratios by TIB quartiles with sleep restfulness. *CI* confidence interval, *IQR* interquartile range, *Q1* lowest quartile, *Q4* highest quartile, *Ref* reference, *TIB* time in bed, *TST* total sleep time.

A regression analysis with TST included as a continuous variable showed a linear trend: as TST increased, all-cause mortality decreased (fully adjusted hazard ratio [HR], 0.996; 95% confidence interval [CI] 0.993–0.999). A linear trend was also observed for TIB; however, this association did not persist after accounting for TST (fully adjusted HR, 1.000; 95% CI 0.997–1.003) (Supplementary Table 1). A regression analysis with TST included as a categorical variable showed that compared to the IQR, the highest TST quartile was consistently associated with lower mortality even after accounting for TIB (fully adjusted HR, 0.50; 95% CI 0.31–0.79). While the lowest TST quartile was associated with higher mortality compared to the IQR, this association became nonsignificant after accounting for TIB (fully adjusted HR, 1.32; 95% CI 0.95–1.84). The highest TIB quartile was also associated with lower mortality relative to the IQR, which, however, did not persist after accounting for TST (fully adjusted HR, 0.71; 95% CI 0.48–1.04) (Table 3; Fig. 1A,B). Results of the sensitivity analysis did not substantially differ (Supplementary Table 2).

**Table 3:** Mortality hazard ratios from cox regression for middle-aged adults (n = 3128).

Predictor	Death rate (%)	Hazard ratio (95% CI)				
		Unadjusted	Age/sex-adjusted	Model 1^a^	Model 2^b^	Model 3^c^
**TST**						
Q1 (< 331 min)	92/776 (11.9)	1.58 (1.20–2.08)	1.54 (1.17–2.02)	1.35 (1.02–1.79)	1.34 (1.01–1.78)	1.32 (0.95–1.84)
IQR (331 to < 414 min)	115/1566 (7.3)	Ref	Ref	Ref	Ref	Ref
Q4 (≥ 414 min)	25/786 (3.2)	0.43 (0.28–0.66)	0.49 (0.32–0.75)	0.50 (0.32–0.77)	0.49 (0.32–0.76)	0.50 (0.31–0.79)
**TIB**						
Q1 (< 400 min)	74/779 (9.5)	1.17 (0.88–1.57)	1.20 (0.90–1.60)	1.04 (0.78–1.40)	1.03 (0.77–1.39)	0.79 (0.55–1.12)
IQR (400 to < 477 min)	122/1562 (7.8)	Ref	Ref	Ref	Ref	Ref
Q4 (≥ 477 min)	36/787 (4.6)	0.57 (0.39–0.83)	0.59 (0.41–0.86)	0.58 (0.40–0.85)	0.59 (0.40–0.85)	0.71 (0.48–1.04)
**TST-restfulness**^**d**^						
Q1 (< 331 min)						
Unrestful	44/372 (11.8)	1.70 (1.16–2.47)	1.66 (1.14–2.43)	1.54 (1.05–2.27)	1.57 (1.06–2.33)	1.54 (1.01–2.33)
Restful	48/404 (11.9)	1.64 (1.36–2.38)	1.65 (1.14–2.40)	1.41 (0.96–2.07)	1.37 (0.93–2.02)	1.34 (0.87–2.05)
IQR (331 to < 414 min)						
Unrestful	47/594 (7.9)	1.15 (0.79–1.67)	1.22 (0.84–1.78)	1.27 (0.87–1.86)	1.28 (0.87–1.88)	1.28 (0.87–1.89)
Restful	68/972 (7.0)	Ref	Ref	Ref	Ref	Ref
Q4 (≥ 414 min)						
Unrestful	8/253 (3.2)	0.44(0.20–0.95)	0.51 (0.24–1.11)	0.53 (0.24–1.16)	0.53 (0.24–1.17)	0.55 (0.24–1.21)
Restful	17/533 (3.2)	0.46 (0.27–0.79)	0.53 (0.31–0.91)	0.56 (0.32–0.95)	0.54 (0.32–0.93)	0.55 (0.32–0.97)
**TIB-restfulness**^**e**^						
Q1 (< 400 min)						
Unrestful	29/297 (9.8)	1.37 (0.88–2.12)	1.43 (0.92–2.22)	1.29 (0.82–2.02)	1.30 (0.83–2.05)	0.96 (0.58–1.59)
Restful	45/482 (9.3)	1.28 (0.88–1.88)	1.32 (0.90–1.94)	1.15 (0.78–1.70)	1.14 (0.77–1.68)	0.86 (0.56–1.34)
IQR (400 to < 477 min)						
Unrestful	56/631 (8.9)	1.30 (0.90–1.86)	1.34 (0.93–1.93)	1.40 (0.97–2.01)	1.41 (0.97–2.04)	1.33 (0.91–1.93)
Restful	66/931 (7.1)	Ref	Ref	Ref	Ref	Ref
Q4 (≥ 477 min)						
Unrestful	13/291 (4.5)	0.64 (0.36–1.17)	0.68 (0.37–1.22)	0.67 (0.37–1.22)	0.67 (0.37–1.22)	0.74 (0.41–1.36)
Restful	23/496 (4.6)	0.64 (0.39–1.03)	0.67 (0.41–1.08)	0.67 (0.41–1.08)	0.67 (0.42–1.09)	0.81 (0.49–1.33)

*CI* confidence interval, *IQR* interquartile range, *Q1* lowest quartile, *Q4* highest quartile, *Ref* reference, *TIB* time in bed, *TST* total sleep time.

^a^Model 1 included age, sex, race (Caucasian vs. other), body mass index, smoking status, apnea hypopnea index with 4% desaturation, sleep time with saturated oxygen below 80%, stroke, myocardial infarction, hypertension, diabetes, and physical functioning standardized score on the Short Form 36 Health Survey.

^b^Model 2 included Model 1 plus antidepressants, benzodiazepines, Epworth Sleepiness Scale score, number of daytime naps per week, weekend-weekday difference in habitual sleep duration, insomnia or poor sleep, and percent time in rapid eye movement sleep.

^c^Model 3 included Model 2 plus TIB in TST/TST-restfulness models or TST in TIB/TIB-restfulness models.

^d^*P* = 0.85 for interaction between categorical TST and sleep restfulness variables.

^e^*P* = 0.91 for interaction between categorical TIB and sleep restfulness variables.

#### Duration and restfulness

The regression analysis of TST-restfulness classifications showed that the highest TST quartile with feeling rested was associated with lower mortality (fully adjusted HR, 0.55; 95% CI 0.32–0.97), whereas the lowest TST quartile with feeling unrested was associated with higher mortality (fully adjusted HR, 1.54; 95% CI 1.01–2.33), compared to the IQR with feeling rested. Meanwhile, the regression analysis of TIB-restfulness classifications showed no significant associations with mortality (Table 3; Fig. 1C,D). Results of the sensitivity analysis did not differ substantially (Supplementary Table 2).

### Older adults

#### Duration

**Figure 2:**
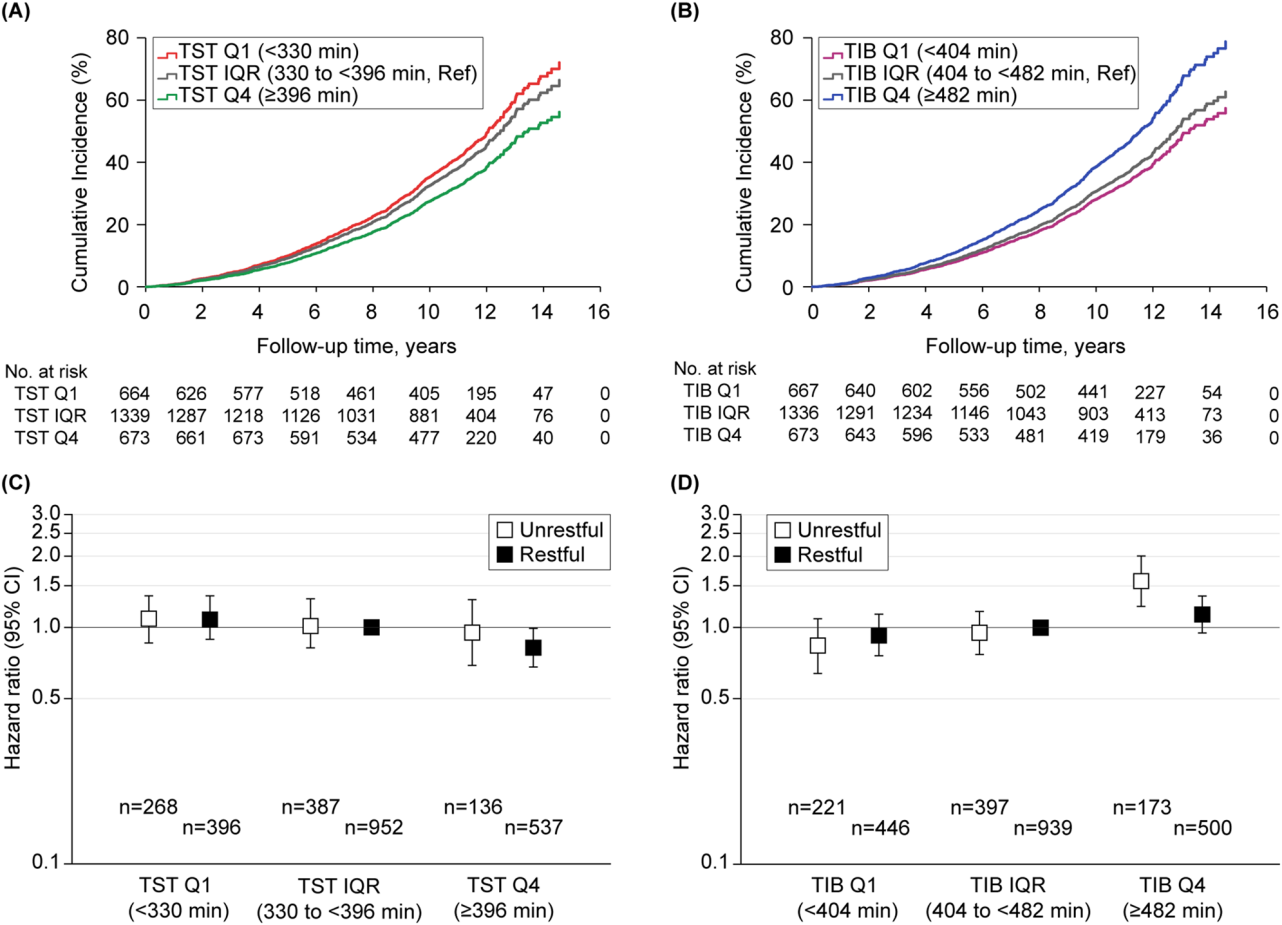
Adjusted Cox regression plots by total sleep time, time in bed, and sleep restfulness for older adults. Differential cumulative incidences or hazard ratios from the fully adjusted Cox proportional hazard models (Model 3) are shown for older adults: (**A**) cumulative incidences by TST quartiles; (**B**) cumulative incidences by TIB quartiles; (**C**) hazard ratios by TST quartiles with sleep restfulness; and (**D**) hazard ratios by TIB quartiles with sleep restfulness. *CI* confidence interval, *IQR* interquartile range, *Q1* lowest quartile, *Q4* highest quartile, *Ref* reference, *TIB* time in bed, *TST* total sleep time.

Similarly as in middle-aged adults, increased TST was linearly associated with decreased all-cause mortality in older adults (fully adjusted HR, 0.998; 95% CI 0.997–0.999). This was not the case for TIB (fully adjusted HR, 1.002; 95% CI 1.000–1.003) (Supplementary Table 1). The regression analysis with TST included as a categorical variable, however, was not significantly associated with mortality. Meanwhile, the highest TIB quartile was consistently associated with higher mortality, compared to the IQR, even after accounting for TST (fully adjusted HR, 1.25; 95% CI 1.08–1.46), which remained significant in the sensitivity analysis (Table 4; Fig. 2A,B; Supplementary Table 3).

**Table 4:** Mortality hazard ratios from cox regression for older adults (n = 2676).

Predictor	Death rate (%)	Hazard ratio (95% CI)				
		Unadjusted	Age/sex-adjusted	Model 1^a^	Model 2^b^	Model 3^c^
**TST**						
Q1 (< 310 min)	299/664 (45.0)	1.17 (1.01–1.34)	1.12 (0.97–1.29)	1.04 (0.90–1.20)	1.01 (0.88–1.17)	1.08 (0.92–1.27)
IQR (310 to < 396 min)	547/1339 (40.9)	Ref	Ref	Ref	Ref	Ref
Q4 (≥ 396 min)	228/673 (33.9)	0.80 (0.68–0.93)	0.90 (0.77–1.05)	0.89 (0.76–1.04)	0.88 (0.75–1.03)	084 (0.72–1.00)
**TIB**						
Q1 (< 404 min)	270/667 (40.5)	1.08 (0.93–1.25)	1.09 (0.94–1.26)	1.07 (0.92–1.24)	1.05 (0.91–1.22)	0.91 (0.76–1.08)
IQR (404 to < 482 min)	508/1336 (38.0)	Ref	Ref	Ref	Ref	Ref
Q4 (≥ 482 min)	296/673 (44.0)	1.25 (1.08–1.44)	1.22 (1.05–1.41)	1.18 (1.02–1.37)	1.18 (1.02–1.36)	1.25 (1.08–1.46)
**TST-restfulness**^**d**^						
Q1 (< 310 min)						
Unrestful	113/268 (42.2)	1.05 (0.85–1.30)	1.10 (0.89–1.36)	1.03 (0.83–1.28)	1.02 (0.82–1.27)	1.09 (0.86–1.36)
Restful	186/396 (47.0)	1.17 (0.98–1.40)	1.11 (0.93–1.33)	1.05 (0.88–1.26)	1.02 (0.82–1.27)	1.08 (0.89–1.32)
IQR (310 to < 396 min)						
Unrestful	145/387 (37.5)	0.87 (0.71–1.05)	0.97 (0.80–1.18)	1.00 (0.82–1.22)	1.01 (0.83–1.23)	1.01 (0.83–1.24)
Restful	401/952 (42.1)	Ref	Ref	Ref	Ref	Ref
Q4 (≥ 396 min)						
Unrestful	52/136 (38.2)	0.89 (0.65–1.20)	1.07 (0.79–1.44)	1.01 (0.74–1.39)	0.99 (0.72–1.36)	0.95 (0.69–1.31)
Restful	176/537 (32.8)	0.73 (0.61–0.88)	0.85 (0.71–1.01)	0.86 (0.71–1.03)	0.86 (0.71–1.03)	0.82 (0.68–0.99)
**TIB-restfulness**^**e**^						
Q1 (< 404 min)						
Unrestful	87/221 (39.4)	1.00 (0.79–1.27)	1.04 (0.82–1.32)	0.99 (0.78–1.26)	0.99 (0.78–1.27)	0.84 (0.64–1.09)
Restful	184/446 (41.3)	1.08 (0.90–1.29)	1.11 (0.93–1.33)	1.10 (0.92–1.32)	1.07 (0.89–1.29)	0.93 (0.76–1.14)
IQR (404 to < 482 min)						
Unrestful	143/397 (36.0)	0.93 (0.76–1.14)	1.00 (0.82–1.23)	0.99 (0.80–1.22)	0.98 (0.80–1.21)	0.95 (0.77–1.17)
Restful	365/939 (38.9)	Ref	Ref	Ref	Ref	Ref
Q4 (≥ 482 min)						
Unrestful	81/173 (46.8)	1.37 (1.07–1.75)	1.51 (1.18–1.93)	1.52 (1.18–1.94)	1.51 (1.17–1.93)	1.57 (1.23–2.01)
Restful	215/500 (43.0)	1.17 (0.99–1.39)	1.13 (0.95–1.34)	1.08 (0.91–1.29)	1.07 (0.90–1.28)	1.14 (0.95–1.36)

*CI* confidence interval, *IQR* interquartile range, *Q1* lowest quartile, *Q4* highest quartile, *Ref* reference, *TIB* time in bed, *TST* total sleep time.

^a^Model 1 included age, sex, race (Caucasian vs. other), body mass index, smoking status, apnea hypopnea index with 4% desaturation, sleep time with saturated oxygen below 80%, stroke, myocardial infarction, hypertension, diabetes, and physical functioning standardized score on the Short Form 36 Health Survey.

^b^Model 2 included Model 1 plus antidepressants, benzodiazepines, Epworth Sleepiness Scale score, number of daytime naps per week, weekend-weekday difference in habitual sleep duration, insomnia or poor sleep, and percent time in rapid eye movement sleep.

^c^Model 3 included Model 2 plus TIB in TST/TST-restfulness models or TST in TIB/TIB-restfulness models.

^d^*P* = 0.22 for interaction between categorical TST and sleep restfulness variables.

^e^ *P* = 0.28 for interaction between categorical TIB and sleep restfulness variables.

#### Duration and restfulness

The regression analysis of TST-restfulness classifications showed that compared to the IQR with feeling rested, the highest TST quartile with feeling rested was partly associated with lower mortality (fully adjusted HR, 0.82; 95% CI 0.68–0.99), which, however, did not persist in the sensitivity analysis. Meanwhile, the regression analysis of TIB-restfulness classifications showed that compared to the IQR with feeling rested, the highest TIB quartile with feeling unrested was consistently associated with higher mortality (fully adjusted HR, 1.57; 95% CI 1.23–2.01), which remained the same in the sensitivity analysis (Table 4; Fig. 2C,D; Supplementary Table 3).

### Complete-case analysis

Among both age groups, the results of the Cox models after imputation were similar to those of the complete-case analysis (Supplementary Tables 4 and 5).

## Discussion

We showed that polysomnography (PSG)-measured TST and TIB at night, alone or together with the sense of feeling rested after sleep, were differentially associated with all-cause mortality in middle-aged and older adults. Although there was no significant interaction between TST or TIB and sleep restfulness, our findings improve our understanding of their interrelationship with respect to mortality outcomes.

The association between PSG-measured sleep duration and all-cause mortality among middle-aged adults was predominantly linear, with longer durations being protective and shorter durations being hazardous. These findings contrast sharply with those from many reports linking subjective long sleep duration to increased mortality^1,27–29^. Kripke et al. associated actigraphic long sleep duration with higher mortality; however, their supplemental analysis showed that TIB was a stronger risk factor for mortality than TST, indicating the possibility that the association between the two stems from other factors^5^. Meanwhile, our findings are in line with evidence linking objective short sleep duration with increased mortality^5–7^, providing further support for the role of sleep in physiological homeostasis. The cumulative effect of not getting enough sleep (sleep debt), which is likely to be greater in middle-aged or younger adults^10,11^, could involve the dysregulation of the autonomic nervous system, metabolic hormones, and inflammation^30–32^, putting people at risk of a variety of medical and psychiatric conditions, ultimately leading to early mortality. Although we adjusted for subjective measures of sleep debt, there remain potential confounders associated with individual differences in objective sleep debt including difference between objective weekday and weekend sleep durations^33^ or those with physiological tolerance to homeostatic sleep need^34,35^.

Notably, our results from joint analyses in middle-aged adults suggest that sleep restfulness could have a role in determining whether a certain amount of sleep is protective or hazardous, providing support for the hypothesis that restorative sleep is a determinant of human health outcomes. Mixed results were obtained with the combined effects of PSG-measured short sleep duration (< 6 h) and insomnia on mortality in community-based studies including the SHHS^6,7^. Given these findings, the current findings improve our understanding of the difference between nonrestorative sleep and other insomnia-related presentations^19,21^, and reveal the potential of sleep restfulness measures in the assessment of longitudinal health outcomes. Meanwhile, in the middle-aged group, TIB-restfulness categories did not capture high-risk populations. Thus, sufficient objective TST and subjective restorative sleep might be vital in promoting health in middle-aged adults.

Contrastingly, among older adults, we showed a hazardous effect of PSG-measured long TIB on all-cause mortality, which is consistent with the observation of Kripke et al. that actigraphic TIB was a stronger risk factor for mortality than actigraphic TST^5^. Importantly, our results showed that when considering sleep restfulness simultaneously, participants who felt unrested despite long TIB had increased mortality. These individual and combined effects of long TIB with sleep restfulness persisted while accounting for both TST and those dying within 2 years, suggesting that neither sleep duration itself nor the factors involved in the dying process might explain an increase in mortality. Further, our findings remained unchanged after accounting for antidepressants or benzodiazepines, which have been suggested to explain the association of long sleep duration with increased mortality^36^.

Daytime resting behavior (bed rest or naps), which could also influence nocturnal sleep, was subjectively assessed but not objectively controlled in the current study. Nevertheless, corroborating previous findings^8^, an age-related decline in TST, but not a decline in TIB, was observed, indicating that a relative excess of TIB may be a byproduct of aging. In accordance with physiological implications that older adults are most likely to experience complications of prolonged bed rest, such as decreased cardiac output, relative hypoxemia, or muscle atrophy^15^, experimental evidence suggests that TIB extension could be hazardous as indicated by increased sleepiness, depression, and inflammation^37^, whereas TIB restriction improves objective sleep continuity and sleep depth among older adults^38–40^. Taken together, longer TIB could be associated with worse health outcomes, whereas it is not clear that longer TST is more protective against mortality in older adults. Furthermore, it is plausible that sleep homeostasis cannot be fulfilled, particularly when nonrestorative sleep coexists with longer TIB.

We did not make an a priori hypothesis on an operational definition of PSG-measured short or long TST or TIB to be adopted, as definitive evidence was unavailable for our study purposes. The decisions taken a priori were to adopt the highest and lowest quartiles of PSG-measured TST or TIB with the IQR serving as the reference category, while eliminating assumptions derived from subjective assessments of sleep habits. Moreover, we defined the cutoff values according to the age categories of our study sample (i.e., middle-aged and older groups) because age considerably affects sleep duration^8^. Interestingly our findings suggested that among middle-aged adults, the mortality rates decreased as TST increased regardless of the cutoff values. Moreover, considering our results obtained from linear regression analyses, we cannot ignore the possibility that the observed association between long TIB and increased mortality depends on how a long TIB is defined among older adults. The issue of operational definition is also the case for sleep restfulness. As we assumed that sleep restfulness might stem from certain physiological processes, we adopted a definition similar to that reported in a previous study, which suggested the association of unrestful sleep with sleep instability (sleep stage transitions)^23^. However, we cannot eliminate the possibility that different cutoffs for sleep restfulness can change our conclusions regarding the joint effects of sleep restfulness with TST or TIB on mortality.

### Strengths and limitations

The strengths of our study include the relatively large sample of middle-aged and older populations with men and women, availability of longitudinal data, objective sleep measures, and the sensitivity analysis conducted to control for reverse causality. Nevertheless, this study also has several limitations. A single-night PSG study could underestimate sleep duration due to an individual’s response to PSG, referred to as the first-night effect. However, a study using the same PSG protocol found no significant differences in sleep duration between two recordings^41^. Another in-home PSG study also showed that both TST and TIB did not significantly differ across multiple consecutive nights^42^. Therefore, our findings could not be fully explained by the first-night effect. We focused on the temporary feeling of rest following the night after PSG, as opposed to the habitual feeling of rest after sleep relied upon by most studies. Although more research is needed to understand the function of temporary sleep restfulness as compared to that of habitual sleep restfulness, measuring temporary sleep restfulness may provide greater reliability, specificity, and feasibility in future epidemiological research. Moreover, prior research that explored objective sleep correlates of subjective sleep restfulness identified its relationship with sleep efficiency or sleep continuity measures^22–24^. Thus, these measures could serve as an intervention target for improving sleep restfulness. As we did not observe the interactive effects between sleep duration measures and sleep restfulness on mortality, possible protective effects of optimization of TST or TIB on mortality by improving sleep restfulness can be the subject of future longitudinal investigations. Although further investigations are needed to determine the public health utility of such combined subjective/objective sleep measures in different community samples, our findings may certainly provide important insights into the association among human nocturnal resting behavior, sleep restfulness, and mortality, and epidemiological implications for adequate sleep hygiene among middle-aged and older adults.

## Methods

### Participants

All data were derived from the SHHS. Details of the study are available elsewhere^25^. The study was performed in accordance with the Helsinki Declaration and each participant provided written informed consent. A total of 6441 participants aged 40 years and older were enrolled from existing cohorts and underwent the baseline examination between 1995 and 1998. Of these, 5804 participants who underwent overnight PSG, comprising 3128 middle-aged (40–64 years) and 2676 older (≥ 65 years) adults, were included in the final dataset. The distinction of middle-aged and older adults relied on the National Sleep Foundation’s expert consensus age categories^43^. The current project was approved in April 2020 by the Ethics Committee of National Center of Neurology and Psychiatry (project number: A2020-012). All analyzed data are publicly available (sleepdata.org).

### Measures

#### Objective sleep measure

An unattended, portable in-home PSG was conducted during the baseline examination using the Compumedics P Series System (Abbotsford, Victoria, Australia). Standard PSG characteristics, including TST (total time in non-rapid eye-movement stages 1–3 and rapid eye-movement sleep) and TIB (time between the electronically marked bedtime and final rising time), were evaluated based on the SHHS Reading Center manual of operations, as described previously^44^.

#### Subjective sleep measure

In the morning following the PSG monitoring, the participants rated the “sleep restfulness” of the previous night’s sleep using a five-point Likert-type scale, with higher scores indicating more restfulness (1 = restless; 5 = restful)^22,23^.

#### Primary exposure

The primary exposures were PSG-measured short and long TST (vs. medium TST), short and long TIB (vs. medium TIB), and feeling unrested after sleep (vs. feeling rested). The three TST and TIB categories and two sleep restfulness categories were combined to generate six TST-restfulness and TIB-restfulness categories, i.e., short-unrested, short-rested, medium-unrested, medium-rested, long-unrested, and long-rested. Short and long durations of TST and TIB were determined based on the lowest (Q1) and highest (Q4) quartiles in each age group, respectively. Regarding sleep restfulness, based on a prior SHHS analysis^23^, a score < 3 on the sleep restfulness scale was defined as feeling unrested, whereas a score ≥ 3 was defined as feeling rested.

#### Mortality outcome

Deaths from any cause were identified using multiple concurrent approaches including follow-up interviews, written annual questionnaires or telephonic conversations with participants or next-of-kin, surveillance of local hospital records and community obituaries, and linkage with the Social Security Administration Death Master File, as described elsewhere^45^.

#### Other covariates

Baseline sociodemographic and health covariates included age, sex, race/ethnicity (Caucasian and other), smoking status (current, former, and never), body mass index, hypertension (defined as an average systolic blood pressure > 140 mmHg or average diastolic blood pressure > 90 mmHg, or the use of antihypertensive medications), diabetes (self-reported or determined by the use of insulin or hypoglycemic medications), stroke and myocardial infarction (identified by a self-reported history of diagnosis by a physician), and physical function levels defined by the physical functioning standardized score on the Short Form 36 Health Survey^46^. Additionally, baseline sleep-related covariates included the daytime sleepiness level defined by the Epworth Sleepiness Scale^47^, difference between self-reported habitual sleep duration at night on weekdays (or workdays) and that on weekends (or non-workdays) (both collected in h), number of naps for 5 min or longer per week, insomnia or poor sleep as indicated by a self-reported consumption of sleeping pills or difficulty in initiating or maintaining sleep^7^, and the use of antidepressants or benzodiazepines.

### Statistical analysis

Of the 5804 individuals analyzed, 824 (26.3%) middle-aged adults and 563 (21.0%) older adults had at least one missing value in the baseline covariates. We replaced the missing data using multiple imputation by chained equations with 20 imputed datasets under the assumption of data missing at random^48,49^. We used Cox proportional hazard models to assess associations between the duration of nighttime resting behavior (TST or TIB), sleep restfulness, and all-cause mortality using our exposure of interest. While we assumed that an intermediate TST or TIB would be associated with the lowest risk of mortality given the available literature^1–3^, another line of evidence suggests that objective sleep duration could be linearly associated with mortality risk, with longer sleep duration being protective against mortality^6,7^. Therefore, we first assessed the individual effect of TST or TIB as a continuous variable on mortality. We then assessed the individual effect of TST or TIB as a categorical variable on mortality. Finally, we assessed the joint effects of TST or TIB and sleep restfulness on mortality. Cox models were run separately for middle-aged and older adults. Results are shown as hazard ratios with 95% confidence intervals. To test for effect modification in each joint analysis, an interaction term between TST or TIB and sleep restfulness was entered into each model. *P* < 0.05 was considered statistically significant. All analyses were performed using SPSS Statistics, version 23 (IBM Japan, Tokyo).

In addition to unadjusted and age/sex-adjusted models, we ran three multivariable-adjusted models. Model 1 included demographic and health covariates selected based on the known risk factors of mortality, including age, sex, race (Caucasian vs. other), body mass index, smoking status, apnea hypopnea index with 4% oxygen desaturation, sleep time with saturated oxygen below 80%, stroke, myocardial infarction, hypertension, diabetes, and the physical functioning standardized score on the Short Form 36 Health Survey. Model 2 further included sleep-related covariates, including the use of antidepressants or benzodiazepines, score on the Epworth Sleepiness Scale, number of daytime naps per week, weekend-weekday difference in habitual sleep duration as an index of potential sleep debt^50^, insomnia or poor sleep^7^, and rapid eye-movement sleep percentage, which has been shown not only to negatively associate with mortality risk in community-based cohorts, including the SHHS^51,52^, but also to be more variable than non-rapid eye-movement sleep stages across in-home PSG nights^41,42^. Model 3 further included TIB in the TST/TST-restfulness models or TST in the TIB/TIB-restfulness models to differentiate the effect of TST from that of TIB, or vice versa.

Finally, we conducted sensitivity analyses by excluding (1) those dying and censored in the first 2 years following baseline to control for the known changes in sleep duration in the last months of life^53^. We compared the complete-case analysis with the results of multiple imputation models^54^.

## Supplementary Information

Supplementary Information.

## Data Availability

The data underlying this article are available in NSRR, at https://sleepdata.org/.

## References

[1] da Silva AA (2016). Sleep duration and mortality in the elderly: A systematic review with meta-analysis. BMJ Open.

[2] Cappuccio FP, D’Elia L, Strazzullo P, Miller MA (2010). Sleep duration and all-cause mortality: A systematic review and meta-analysis of prospective studies. Sleep.

[3] Aurora RN, Kim JS, Crainiceanu C, O’Hearn D, Punjabi NM (2016). Habitual sleep duration and all-cause mortality in a general community sample. Sleep.

[4] Lecci S (2020). Electroencephalographic changes associated with subjective under- and overestimation of sleep duration. Sleep.

[5] Kripke DF, Langer RD, Elliott JA, Klauber MR, Rex KM (2011). Mortality related to actigraphic long and short sleep. Sleep Med..

[6] Vgontzas AN (2010). Insomnia with short sleep duration and mortality: The Penn State cohort. Sleep.

[7] Bertisch SM (2018). Insomnia with objective short sleep duration and risk of incident cardiovascular disease and all-cause mortality: Sleep Heart Health Study. Sleep.

[8] Ohayon MM, Carskadon MA, Guilleminault C, Vitiello MV (2004). Meta-analysis of quantitative sleep parameters from childhood to old age in healthy individuals: Developing normative sleep values across the human lifespan. Sleep.

[9] Skorucak J, Arbon EL, Dijk DJ, Achermann P (2018). Response to chronic sleep restriction, extension, and subsequent total sleep deprivation in humans: Adaptation or preserved sleep homeostasis?. Sleep.

[10] Dijk DJ, Groeger JA, Stanley N, Deacon S (2010). Age-related reduction in daytime sleep propensity and nocturnal slow wave sleep. Sleep.

[11] Fox EC (2018). Sleep debt at the community level: Impact of age, sex, race/ethnicity and health. Sleep Health.

[12] Youngstedt SD, Kripke DF (2004). Long sleep and mortality: Rationale for sleep restriction. Sleep Med. Rev..

[13] Klerman EB, Dijk DJ (2008). Age-related reduction in the maximal capacity for sleep—Implications for insomnia. Curr. Biol..

[14] Grandner MA, Drummond SP (2007). Who are the long sleepers? Towards an understanding of the mortality relationship. Sleep Med. Rev..

[15] Harper CM, Lyles YM (1988). Physiology and complications of bed rest. J. Am. Geriatr. Soc..

[16] Li Y (2014). Association between insomnia symptoms and mortality: A prospective study of US men. Circulation.

[17] Ricardo AC (2017). Association of sleep duration, symptoms, and disorders with mortality in adults with chronic kidney disease. Kidney Int. Rep..

[18] Laugsand LE, Strand LB, Vatten LJ, Janszky I, Bjørngaard JH (2014). Insomnia symptoms and risk for unintentional fatal injuries-The HUNT study. Sleep.

[19] Zhang J (2013). Differentiating nonrestorative sleep from nocturnal insomnia symptoms: Demographic, clinical, inflammatory, and functional correlates. Sleep.

[20] Ohayon MM (2005). Prevalence and correlates of nonrestorative sleep complaints. Arch. Intern. Med..

[21] Wilkinson K, Shapiro C (2012). Nonrestorative sleep: Symptom or unique diagnostic entity?. Sleep Med..

[22] Kaplan KA, Hardas PP, Redline S, Zeitzer JM, Sleep Heart Health Study Research Group (2017). Correlates of sleep quality in midlife and beyond: a machine learning analysis. Sleep Med..

[23] Caffo B, Swihart BJ, Punjabi NM (2010). Utility of sleep stage transitions in assessing sleep continuity. Sleep.

[24] Kaplan KA (2017). When a gold standard isn’t so golden: Lack of prediction of subjective sleep quality from sleep polysomnography. Biol. Psychol..

[25] Quan SF (1997). The Sleep Heart Health Study: Design, rationale, and methods. Sleep.

[26] Zhang GQ (2018). The National Sleep Research Resource: Towards a sleep data commons. J. Am. Med. Inform. Assoc..

[27] Tamakoshi A, Ohno Y, JACC Study Group (2004). Self-reported sleep duration as a predictor of all-cause mortality: Results from the JACC study, Japan. Sleep.

[28] Shen X, Wu Y, Zhang D (2016). Nighttime sleep duration, 24-h sleep duration and risk of all-cause mortality among adults: A meta-analysis of prospective cohort studies. Sci. Rep..

[29] Kwok CS (2018). Self-reported sleep duration and quality and cardiovascular disease and mortality: A dose-response meta-analysis. J. Am. Heart Assoc..

[30] Tobaldini E (2019). Short sleep duration and cardiometabolic risk: From pathophysiology to clinical evidence. Nat. Rev. Cardiol..

[31] Irwin MR, Olmstead R, Carroll JE (2016). Sleep disturbance, sleep duration, and inflammation: A systematic review and meta-analysis of cohort studies and experimental sleep deprivation. Biol. Psychiatry.

[32] Kitamura S (2016). Estimating individual optimal sleep duration and potential sleep debt. Sci. Rep..

[33] Åkerstedt T (2019). Sleep duration and mortality—Does weekend sleep matter?. J. Sleep Res..

[34] Aeschbach D (2001). Evidence from the waking electroencephalogram that short sleepers live under higher homeostatic sleep pressure than long sleepers. Neuroscience.

[35] Aeschbach D, Cajochen C, Landolt H, Borbély AA (1996). Homeostatic sleep regulation in habitual short sleepers and long sleepers. Am. J. Physiol..

[36] Patel SR, Malhotra A, Gottlieb DJ, White DP, Hu FB (2006). Correlates of long sleep duration. Sleep.

[37] Reynold AM, Bowles ER, Saxena A, Fayad R, Youngstedt SD (2014). Negative effects of time in bed extension: A pilot study. J. Sleep Med. Disord..

[38] Youngstedt SD (2009). Tolerance of chronic 90-min time-in-bed restriction in older long sleepers. Sleep.

[39] Hoch CC (2001). Protecting sleep quality in later life: A pilot study of bed restriction and sleep hygiene. J. Gerontol. B Psychol. Sci. Soc. Sci..

[40] Zielinski MR, Kline CE, Kripke DF, Bogan RK, Youngstedt SD (2008). No effect of 8-week time in bed restriction on glucose tolerance in older long sleepers. J. Sleep Res..

[41] Quan SF (2002). Short-term variability of respiration and sleep during unattended nonlaboratory polysomnography—The sleep heart health study. Sleep.

[42] Le Bon O (2001). The first-night effect may last more than one night. J. Psychiatr. Res..

[43] Hirshkowitz M (2015). National sleep foundation’s sleep time duration recommendations: Methodology and results summary. Sleep Heal..

[44] Redline S (1998). Methods for obtaining and analyzing unattended polysomnography data for a multicenter study. Sleep Heart Health Research Group. Sleep.

[45] Punjabi NM (2009). Sleep-disordered breathing and mortality: A prospective cohort study. PLoS Med..

[46] Ware JE, Sherbourne CD (1992). The MOS 36-ltem Short-Form Health Survey (SF-36). I. Conceptual framework and item selection. Med. Care.

[47] Johns MW (1992). Reliability and factor analysis of the Epworth Sleepiness Scale. Sleep.

[48] White IR, Royston P, Wood AM (2011). Multiple imputation using chained equations: Issues and guidance for practice. Stat. Med..

[49] Sterne JA (2009). Multiple imputation for missing data in epidemiological and clinical research: Potential and pitfalls. BMJ.

[50] Cabeza de Baca T (2019). Sleep debt: The impact of weekday sleep deprivation on cardiovascular health in older women. Sleep.

[51] Leary EB (2020). Association of rapid eye movement sleep with mortality in middle-aged and older adults. JAMA Neurol..

[52] Zhang J (2019). Influence of rapid eye movement sleep on all-cause mortality: A community-based cohort study. Aging (Albany NY).

[53] Teno JM, Weitzen S, Fennell ML, Mor V (2001). Dying trajectory in the last year of life: Does cancer trajectory fit other diseases?. J. Palliat. Med..

[54] White IR, Carlin JB (2010). Bias and efficiency of multiple imputation compared with complete-case analysis for missing covariate values. Stat. Med..

