# Supplementary material for: Mortality associated with nonrestorative short sleep or nonrestorative long time-in-bed in middle-aged and older adults
Source: Sci Rep. 2022 Jan 7;12:189. doi: 10.1038/s41598-021-03997-z (PMC8741976; doi:10.1038/s41598-021-03997-z)
Supplement: Supplementary file 1 — Supplementary Information. [file 41598_2021_3997_MOESM1_ESM.docx]

**Supplementary Material**

**Mortality associated with nonrestorative short sleep or nonrestorative long time-in-bed in middle-aged and older adults**

**Supplementary Fig. 1**. Age-related Changes in Resting Behavior and Sleep Restfulness

**Supplementary Table 1**. Mortality Hazard Ratios from Cox Regression of Total Sleep Time or Time in Bed as A Continuous Variable in Middle-aged (n = 3128) and Older Adults (n = 2676)

**Supplementary Table 2**. Sensitivity Analysis of Mortality Hazard Ratios from Cox Regression in Middle-aged Adults (n = 3083)

**Supplementary Table 3**. Sensitivity Analysis of Mortality Hazard Ratios from Cox Regression in Older Adults (n = 2574)

**Supplementary Table 4**. Mortality Hazard Ratios from Complete-Case Analysis in Middle-aged Adults

**Supplementary Table 5**. Mortality Hazard Ratios from Complete-Case Analysis in Older Adults

**Supplementary Fig. 1. Age-related Changes in Resting Behavior and Sleep Restfulness**

**

**

Error bars represent 95% confidence intervals.

**Supplementary Table 1. Mortality Hazard Ratios from Cox Regression of Total Sleep Time or Time in Bed as A Continuous Variable in Middle-aged (n = 3128) and Older Adults (n = 2676)**

| **Predictor** | **Death rate (%)** | **Hazard Ratio (95% CI)** | | | | |
| --- | --- | --- | --- | --- | --- | --- |
|  |  | **Unadjusted** | **Age/sex-adjusted** | **Model 1^a^** | **Model 2^b^** | **Model 3^c^** |
| Middle-aged adults |  |  |  |  |  |  |
| TST | 232/3128 (7.4) | 0.994 (0.992–0.996) | 0.995 (0.993–0.997) | 0.996 (0.994–0.998) | 0.996 (0.994–0.998) | 0.996 (0.993–0.999) |
| TIB | 232/3128 (7.4) | 0.996 (0.994–0.998) | 0.996 (0.994–0.998) | 0.997 (0.995–0.999) | 0.997 (0.995–0.999) | 1.000 (0.997–1.003) |
| Older adults |  |  |  |  |  |  |
| TST | 1074/2676 (40.1) | 0.998 (0.997–0.999) | 0.998 (0.997–0.999) | 0.999 (0.998–1.000) | 0.999 (0.998–1.000) | 0.998 (0.997–0.999) |
| TIB | 1074/2676 (40.1) | 1.000 (0.999–1.001) | 1.000 (0.999–1.001) | 1.000 (0.999–1.001) | 1.000 (0.999–1.001) | 1.002 (1.000–1.003) |

^a^ Model 1 included age, sex, race (Caucasian vs. other), body mass index, smoking status, apnea hypopnea index with 4% desaturation, sleep time with saturated oxygen below 80%, stroke, myocardial infarction, hypertension, diabetes, and physical functioning standardized score on the Short Form 36 Health Survey. ^b^ Model 2 included Model 1 plus the use of antidepressants and benzodiazepines, Epworth sleepiness scale score, number of daytime naps per week, weekend-weekday difference in habitual sleep duration, insomnia or poor sleep, and percent time in rapid eye movement sleep. ^c^ Model 3 included Model 2 plus TIB in TST/TST-restfulness models or TST in TIB/TIB-restfulness models.

CI, confidence interval; TST, total sleep time; TIB, time in bed.

**Supplementary Table 2. Sensitivity Analysis of Mortality Hazard Ratios from Cox Regression in Middle-aged Adults (n = 3083)**

| **Predictor** | **Death rate (%)** | **Hazard Ratio (95% CI)** | | | | |
| --- | --- | --- | --- | --- | --- | --- |
|  |  | **Unadjusted** | **Age/sex-adjusted** | **Model 1^a^** | **Model 2^b^** | **Model 3^c^** |
| TST |  |  |  |  |  |  |
| Q1 (<331 min) | 88/769 (11.4) | 1.64 (1.24–2.18) | 1.59 (1.20–2.12) | 1.40 (1.04–1.86) | 1.38 (1.03–1.85) | 1.36 (0.97–1.91) |
| IQR (331 to <414 min) | 106/1541 (6.9) | Ref | Ref | Ref | Ref | Ref |
| Q4 (≥414 min) | 23/773 (3.0) | 0.43 (0.27–0.67) | 0.48 (0.31–0.76) | 0.50 (0.32–0.78) | 0.49 (0.31–0.78) | 0.39 (0.23–0.67) |
| TIB |  |  |  |  |  |  |
| Q1 (<400 min) | 69/764 (9.0) | 1.18 (0.87–1.59) | 1.21 (0.89–1.63) | 1.05 (0.77–1.42) | 1.04 (0.76–1.41) | 0.77 (0.54–1.11) |
| IQR (400 to <478 min) | 115/1548 (7.4) | Ref | Ref | Ref | Ref | Ref |
| Q4 (≥478 min) | 33/771 (4.3) | 0.56 (0.38–0.83) | 0.58 (0.39–0.86) | 0.57 (0.38–0.84) | 0.57 (0.39–0.85) | 0.70 (0.46–1.05) |
| TST-Restfulness |  |  |  |  |  |  |
| Q1 (<331 min) |  |  |  |  |  |  |
| Unrestful | 42/368 (11.4) | 1.78 (1.20–2.64) | 1.75 (1.18–2.59) | 1.61 (1.08–2.40) | 1.62 (1.08–2.43) | 1.58 (1.03–2.44) |
| Restful | 46/401 (11.5) | 1.74 (1.19–2.56) | 1.76 (1.20–2.58) | 1.52 (1.03–2.25) | 1.48 (1.00–2.20) | 1.44 (0.92–2.24) |
| IQR (331 to <414 min) |  |  |  |  |  |  |
| Unrestful | 44/586 (7.5) | 1.20 (0.81–1.77) | 1.28 (0.86–1.89) | 1.33 (0.90–1.98) | 1.33 (0.90–2.00) | 1.34 (0.90–2.00) |
| Restful | 62/955 (6.5) | Ref | Ref | Ref | Ref | Ref |
| Q4 (≥414 min) |  |  |  |  |  |  |
| Unrestful | 7/249 (2.8) | 0.43 (0.19–0.97) | 0.50 (0.22–1.14) | 0.52 (0.23–1.18) | 0.52 (0.23–1.19) | 0.53 (0.23–1.24) |
| Restful | 16/524 (3.1) | 0.47 (0.27–0.83) | 0.54 (0.31–0.95) | 0.57 (0.33–1.00) | 0.56 (0.32–0.98) | 0.57 (0.32–1.03) |

^a^ Model 1 included age, sex, race (Caucasian vs. other), body mass index, smoking status, apnea hypopnea index with 4% desaturation, sleep time with saturated oxygen below 80%, stroke, myocardial infarction, hypertension, diabetes, and physical functioning standardized score on the Short Form 36 Health Survey. ^b^ Model 2 included Model 1 plus the use of antidepressants and benzodiazepines, Epworth sleepiness scale score, number of daytime naps per week, weekend-weekday difference in habitual sleep duration, insomnia or poor sleep, and percent time in rapid eye movement sleep. ^c^ Model 3 included Model 2 plus TIB in TST/TST-restfulness models or TST in TIB/TIB-restfulness models.

CI, confidence interval; IQR, interquartile range; Q1, lowest quartile; Q4, highest quartile; Ref, reference; TIB, time in bed; TST, total sleep time.**Supplementary Table 3. Sensitivity Analysis of Mortality Hazard Ratios from Cox Regression in Older Adults (n = 2574)**

| **Predictor** | **Death rate (%)** | **Hazard Ratio (95% CI)** | | | | |
| --- | --- | --- | --- | --- | --- | --- |
|  |  | **Unadjusted** | **Age/sex-adjusted** | **Model 1^a^** | **Model 2^b^** | **Model 3^c^** |
| TIB |  |  |  |  |  |  |
| Q1 (<404 min) | 245/640 (38.3) | 1.07 (0.91–1.24) | 1.08 (0.93–1.26) | 1.06 (0.91–1.24) | 1.05 (0.90–1.23) | 0.94 (0.78–1.12) |
| IQR (404 to <481 min) | 465/1291 (36.0) | Ref | Ref | Ref | Ref | Ref |
| Q4 (≥481 min) | 267/643 (41.5) | 1.24 (1.06–1.44) | 1.21 (1.04–1.41) | 1.18 (1.01–1.37) | 1.17 (1.00–1.36) | 1.23 (1.05–1.44) |
| TST-Restfulness |  |  |  |  |  |  |
| Q1 (<310 min) |  |  |  |  |  |  |
| Unrestful | 102/258 (39.5) | 1.01 (0.81–1.27) | 1.05 (0.84–1.31) | 0.99 (0.79–1.25) | 1.00 (0.79–1.26) | 1.04 (0.82–1.33) |
| Restful | 169/385 (43.9) | 1.13 (0.94–1.35) | 1.08 (0.90–1.29) | 1.02 (0.84–1.23) | 1.00 (0.83–1.20) | 1.05 (0.85–1.29) |
| IQR (310 to <397 min) |  |  |  |  |  |  |
| Unrestful | 128/370 (34.6) | 0.84 (0.68–1.03) | 0.96 (0.78–1.17) | 1.00 (0.82–1.23) | 1.01 (0.82–1.25) | 1.02 (0.83–1.25) |
| Restful | 368/918 (40.1) | Ref | Ref | Ref | Ref | Ref |
| Q4 (≥397 min) |  |  |  |  |  |  |
| Unrestful | 50/129 (38.8) | 0.97 (0.71–1.31) | 1.14 (0.83–1.55) | 1.08 (0.77–1.50) | 1.06 (0.76–1.47) | 1.02 (0.73–1.43) |
| Restful | 160/514 (31.1) | 0.74 (0.62–0.90) | 0.86 (0.71–1.04) | 0.86 (0.71–1.04) | 0.86 (0.71–1.05) | 0.84 (0.69–1.02) |
| TIB-Restfulness |  |  |  |  |  |  |
| Q1 (<404 min) |  |  |  |  |  |  |
| Unrestful | 82/215 (38.1) | 1.03 (0.80–1.31) | 1.07 (0.84–1.36) | 1.03 (0.81–1.33) | 1.04 (0.81–1.34) | 0.91 (0.69–1.20) |
| Restful | 163/425 (38.4) | 1.04 (0.86–1.25) | 1.07 (0.89–1.30) | 1.06 (0.87–1.29) | 1.03 (0.85–1.26) | 0.92 (0.74–1.15) |
| IQR (404 to <481 min) |  |  |  |  |  |  |
| Unrestful | 127/380 (33.4) | 0.89 (0.73–1.10) | 0.97 (0.79–1.20) | 0.97 (0.77–1.21) | 0.96 (0.77–1.20) | 0.94 (0.75–1.17) |
| Restful | 338/911 (37.1) | Ref | Ref | Ref | Ref | Ref |
| Q4 (≥481 min) |  |  |  |  |  |  |
| Unrestful | 71/162 (43.8) | 1.31 (1.00–1.70) | 1.45 (1.12–1.89) | 1.47 (1.13–1.91) | 1.47 (1.12–1.92) | 1.52 (1.16–1.98) |
| Restful | 196/481 (40.7) | 1.16 (0.97–1.39) | 1.13 (0.95–1.35) | 1.08 (0.90–1.29) | 1.07 (0.89–1.28) | 1.12 (0.93–1.35) |

^a^ Model 1 included age, sex, race (Caucasian vs. other), body mass index, smoking status, apnea hypopnea index with 4% desaturation, sleep time with saturated oxygen below 80%, stroke, myocardial infarction, hypertension, diabetes, and physical functioning standardized score on the Short Form 36 Health Survey. ^b^ Model 2 included Model 1 plus the use of antidepressants and benzodiazepines, Epworth sleepiness scale score, number of daytime naps per week, weekend-weekday difference in habitual sleep duration, insomnia or poor sleep, and percent time in rapid eye movement sleep. ^c^ Model 3 included Model 2 plus TIB in TST/TST-restfulness models or TST in TIB/TIB-restfulness models.

CI, confidence interval; IQR, interquartile range; Q1, lowest quartile; Q4, highest quartile; Ref, reference; TIB, time in bed; TST, total sleep time.

**Supplementary Table 4. Mortality Hazard Ratios from Complete-Case Analysis in Middle-aged Adults**

| **Predictor** | **Hazard Ratio (95% CI)** | | |
| --- | --- | --- | --- |
|  | **Model 1^a^** | **Model 2^b^** | **Model 3^c^** |
| TST | n=2547 | n=2382 | n=2382 |
| Q1 (<331 min) | 1.38 (1.01–1.88) | 1.35 (0.97–1.88) | 1.27 (0.86–1.87) |
| IQR (331 to <414 min) | Ref | Ref | Ref |
| Q4 (≥414 min) | 0.52 (0.33–0.84) | 0.52 (0.31–0.86) | 0.54 (0.32–0.93) |
| TIB | n=2547 | n=2382 | n=2382 |
| Q1 (<400 min) | 0.99 (0.71–1.37) | 1.08 (0.77–1.53) | 0.81 (0.54–1.22) |
| IQR (400 to <477 min) | Ref | Ref | Ref |
| Q4 (≥477 min) | 0.53 (0.35–0.82) | 0.59 (0.38–0.91) | 0.71 (0.45–1.12) |
| TST-Restfulness | n=2461 | n=2304 | n=2304 |
| Q1 (<331 min) |  |  |  |
| Unrestful | 1.59 (1.04–2.42) | 1.66 (1.05–2.62) | 1.55 (0.95–2.52) |
| Restful | 1.39 (0.91–2.11) | 1.32 (0.83–2.10) | 1.21 (0.72–2.02) |
| IQR (331 to <414 min) |  |  |  |
| Unrestful | 1.19 (0.78–1.83) | 1.38 (0.88–2.17) | 1.40 (0.89–2.20) |
| Restful | Ref | Ref | Ref |
| Q4 (≥414 min) |  |  |  |
| Unrestful | 0.40 (0.16–1.00) | 0.45 (0.18–1.14) | 0.49 (0.19–1.26) |
| Restful | 0.62 (0.35–1.11) | 0.65 (0.35–1.18) | 0.69 (0.37–1.30) |
| TIB-Restfulness | n=2461 | n=2304 | n=2304 |
| Q1 (<400 min) |  |  |  |
| Unrestful | 1.23(0.75–2.03) | 1.52 (0.90–2.57) | 1.11 (0.62–2.01) |
| Restful | 1.09 (0.71–1.68) | 1.21 (0.76–1.93) | 0.93 (0.55–1.56) |
| IQR (400 to <477 min) |  |  |  |
| Unrestful | 1.40 (0.94–2.08) | 1.56 (1.01–2.40) | 1.48 (0.96–2.30) |
| Restful | Ref | Ref | Ref |
| Q4 (≥477 min) |  |  |  |
| Unrestful | 0.49 (0.23–1.03) | 0.58 (0.27–1.25) | 0.65 (0.30–1.40) |
| Restful | 0.73 (0.44–1.24) | 0.82 (0.47–1.42) | 0.98 (0.56–1.74) |

^a^ Model 1 included age, sex, race (Caucasian vs. other), body mass index, smoking status, apnea hypopnea index with 4% desaturation, sleep time with saturated oxygen below 80%, stroke, myocardial infarction, hypertension, diabetes, and physical functioning standardized score on the Short Form 36 Health Survey. ^b^ Model 2 included Model 1 plus the use of antidepressants and benzodiazepines, Epworth sleepiness scale score, number of daytime naps per week, weekend-weekday difference in habitual sleep duration, insomnia or poor sleep, and percent time in rapid eye movement sleep. ^c^ Model 3 included Model 2 plus TIB in TST/TST-restfulness models or TST in TIB/TIB-restfulness models.

CI, confidence interval; IQR, interquartile range; Q1, lowest quartile; Q4, highest quartile; Ref, reference; TIB, time in bed; TST, total sleep time.

**Supplementary Table 5. Mortality Hazard Ratios from Complete-Case Analysis in Older Adults**

| **Predictor** | **Hazard Ratio (95% CI)** | | |
| --- | --- | --- | --- |
|  | **Model 1^a^** | **Model 2^b^** | **Model 3^c^** |
| TST | n=2485 | n=2230 | n=2230 |
| Q1 (<310 min) | 1.03 (0.88–1.19) | 0.98 (0.83–1.15) | 1.03 (0.86–1.23) |
| IQR (310 to <396 min) | Ref | Ref | Ref |
| Q4 (≥396 min) | 0.89 (0.76–1.04) | 0.90 (0.76–1.06) | 0.87 (0.72–1.04) |
| TIB | n=2485 | n=2230 | n=2230 |
| Q1 (<404 min) | 1.05 (0.90–1.22) | 1.00 (0.85–1.19) | 0.90 (0.74–1.09) |
| IQR (404 to <482 min) | Ref | Ref | Ref |
| Q4 (≥482 min) | 1.16 (1.00–1.34) | 1.13 (0.96–1.32) | 1.18 (1.00–1.40) |
| TST-Restfulness | n=2485 | n=2113 | n=2113 |
| Q1 (<310 min) |  |  |  |
| Unrestful | 1.01 (0.80–1.27) | 0.99 (0.77–1.27) | 1.02 (0.79–1.33) |
| Restful | 1.05 (0.87–1.27) | 1.01 (0.82–1.25) | 1.05 (0.84–1.32) |
| IQR (310 to <396 min) |  |  |  |
| Unrestful | 1.00 (0.81–1.23) | 1.06 (0.85–1.32) | 1.06 (0.85–1.32) |
| Restful | Ref | Ref | Ref |
| Q4 (≥396 min) |  |  |  |
| Unrestful | 1.00 (0.72–1.38) | 1.01 (0.71–1.44) | 0.98 (0.68–1.41) |
| Restful | 0.86 (0.71–1.04) | 0.88 (0.72–1.08) | 0.86(0.69–1.06) |
| TIB-Restfulness | n=2345 | n=2113 | n=2113 |
| Q1 (<404 min) |  |  |  |
| Unrestful | 0.95 (0.74–1.23) | 0.95 (0.72–1.25) | 0.83 (0.61–1.11) |
| Restful | 1.05 (0.87–1.28) | 1.00 (0.81–1.24) | 0.89 (0.70–1.13) |
| IQR (404 to <482 min) |  |  |  |
| Unrestful | 0.96 (0.78–1.19) | 0.97 (0.78–1.21) | 0.94 (0.75–1.18) |
| Restful | Ref | Ref | Ref |
| Q4 (≥482 min) |  |  |  |
| Unrestful | 1.42 (1.09–1.85) | 1.46 (1.10–1.94) | 1.51 (1.14–2.00) |
| Restful | 1.02 (0.85–1.22) | 0.99 (0.81–1.20) | 1.04 (0.85–1.27) |

^a^ Model 1 included age, sex, race (Caucasian vs. other), body mass index, smoking status, apnea hypopnea index with 4% desaturation, sleep time with saturated oxygen below 80%, stroke, myocardial infarction, hypertension, diabetes, and physical functioning standardized score on the Short Form 36 Health Survey. ^b^ Model 2 included Model 1 plus the use of antidepressants and benzodiazepines, Epworth sleepiness scale score, number of daytime naps per week, weekend-weekday difference in habitual sleep duration, insomnia or poor sleep, and percent time in rapid eye movement sleep. ^c^ Model 3 included Model 2 plus TIB in TST/TST-restfulness models or TST in TIB/TIB-restfulness models.

CI, confidence interval; IQR, interquartile range; Q1, lowest quartile; Q4, highest quartile; Ref, reference; TIB, time in bed; TST, total sleep time.
